# Screening for cervical cancer in imprisoned women in Brazil

**DOI:** 10.1371/journal.pone.0187873

**Published:** 2017-12-18

**Authors:** Elaine Regina Prudêncio da Silva, Albert Schiaveto de Souza, Taiana Gabriela Barbosa de Souza, Daniel Henrique Tsuha, Ana Rita Barbieri

**Affiliations:** 1 Foundation Federal University of Mato Grosso do Sul, Campo Grande, Mato Grosso do Sul, Brazil; 2 State Health Department of Mato Grosso do Sul, Campo Grande, Mato Grosso do Sul,Brazil; University of Louisville School of Medicine, UNITED STATES

## Abstract

**Context and objective:**

Incarcerated women are more vulnerable to developing cervical cancer than women in general; therefore, screening and intervention programs must be included in their healthcare provision. We therefore aimed to investigate the state of cervical cancer screening for imprisoned women in Mato Grosso do Sul, and to analyze the interventions geared toward the control of cervical cancer.

**Materials and methods:**

This was a cross-sectional study with analysis of primary and secondary data. Interviews were held with 510 women in seven prisons in the Brazilian state of Mato Grosso do Sul. The data for 352 medical records were analyzed statistically with the significance level set at 5%. Associations were assessed by the chi-squared test, adjusted by the Bonferroni correction.

**Results:**

Most female prisoners had limited education, used tobacco, and had key risk factors for the development of cervical cancer. Half of the women interviewed (n = 255) stated that they had received a Papanicolaou (Pap) test in prison, but 134 (52.5%) of these did not know the result. Of those who had not received a Pap test, 149 (58.4%) stated that this was because of a lack of opportunity. There was no information regarding the provision of Pap tests or subsequent treatment in the medical records of 211 (59.9%) women. No protocols were in place for the provision of Pap tests in prison. There were statistical differences between prisons in terms of test frequency, the information provided to women, and how information was recorded in medical records.

**Conclusion:**

The screening of cervical cancer in prisons is neither systematic nor regular, and the results are not communicated to women in a significant number of cases. It is necessary to organize health services within the prison environment, ensuring that tests are done and that there is investigation for human papillomavirus. This could increase the diagnosis of cervical cancer at less advanced stages of the disease.

## Introduction

In 2014, Brazil had the first-largest prison population in the South America 622,202 people. In the world, putting it ahead of India, Thailand, Mexico, Iran, Turkey, and Indonesia. The rate of imprisonment is also rising annually by 7% in Brazil, and at present, 306 people are imprisoned per 100,000 inhabitants, compared with a worldwide rate of approximately 144 people per 100,000 inhabitants [1,2].

Within the prison population, female prisoners area special group because of sex and gender inequalities. Although they represent a minority group, composing 2% to 9% of the world’s prison population, the number of women in prisons is increasing. Indeed, the growth rate is higher than that in the male prison population [3]. Worldwide, more than 700,000 women are imprisoned. Between 2000 and 2014, Brazil recorded an increase of 567% in the female prison population, with some 37,308 Brazilian women being imprisoned in 2014 alone. Brazil now has the fifth-largest population of imprisoned women worldwide [4,5].

In 2016, the state of Mato Grosso do Sul had a prison population in medium- or maximum-security prisons of 12,045 people, of whom 11,041 were male and 1,004 were female; women therefore accounted for 8.34% of prisoners serving their sentences in one of seven prison units in the municipalities of Campo Grande, Corumbá, Jateí, Ponta Porã, Rio Brilhante, São Gabriel do Oeste and TrêsLagoas [6].

Incarcerated women are vulnerable to developing illnesses and health problems for several reasons. These include the prison conditions themselves, such as the overcrowding and associated violence, their social profile, and established behavioral factors that remain after being imprisoned. Important behavioral factors include low educational level, low socioeconomic level, early coitarche, multiplicity of sexual partners, smoking, sexual abuse and violence, illicit drug use, sporadic condom use, and poor access to health actions and health services [3,7]. These characteristics of the female prison population lead to greater susceptibility to sexually transmitted infections. However, emphasis is typically placed on vulnerability to cervical cancer, the main risk factor being the persistence of human papilloma virus (HPV) [8,9].

Cervical cancer is as an important public health problem with high incidence and mortality rates. Worldwide, it is the fourth most common type of cancer among women, but it is the third most frequent in Brazil. In total, 16,340 new cases were recorded in 2016, with an estimated risk of 15.85 new cases per 100,000 women. Because the disease develops slowly and has a long phase before becoming invasive it is suitable for screening, early detection, and treatment if diagnosed in the initial phases [10,11].

As in other countries, the strategy used for cervical cancer screening in Brazil is the Papanicolaou (Pap) test. In Brazil, screening of sexually active women is recommended from the age of 25 through to 64 years old, and further testing can stop at 64 years old if two tests have yielded negative results in the preceding five years. The interval between tests should be three years after two negative results in consecutive years [12–13]. In the USA, the recommendation is to undertake the Pap test in women aged 21–65 years every three years, but women aged 30–65 years can increase the interval between tests from 3 to 5 years if they combine the smear test with the test for detecting HPV [13,14].

To ensure equity in Brazil, screening is recommended for all women, including those in prison. Studies show that this population typically presents high rates of cellular abnormalities [3], with women aged 40 years and older having a risk of cervical cancer four to five times greater than that in women who are not imprisoned. Furthermore, studies show that there can be marked differences in service provision between prisons with discontinuity of health services being common. This led to the need to establish a consensus policy between prisons and health services supported by national directives guaranteeing necessary actions and measures to make the screening, control, and treatment of cervical cancer viable among imprisoned women [3,15,16]. Therefore, in 2014 a joint decision was reached between health and justice institutions in Brazil, which required municipalities to be responsible for healthcare provision in prisons [17]. Since then, health actions and services must be planned and organized by municipalities.

In relation to the female prison population, predisposing factors for cervical cancer are common in their profiles, emphasizing the need to implement and improve strategies that ensure access to strategies for disease control. We aimed to investigate the state of cervical cancer screening and of the interventions geared toward its control among imprisoned women in Mato Grosso do Sul. It was anticipated that the results would allow reflection on the care provision and network for people affected by cervical cancer.

## Materials and methods

### Ethical aspects

The study was approved by the Committee for Ethics in Research with Human Beings of the Federal University of Mato Grosso do Sul under Opinion N.1.250.015 and Certificate of Ethical Appreciation N.49273915.2.0000.0021, which allowed it to be done in conformity with Resolution N.466/2012 of the National Health Council [18]. It was also authorized by the State Agency for the Administration of the Penitentiary System, which is responsible for prisons in Mato Grosso do Sul. To ensure imprisoned women participated freely, guidance was provided to each of the prison governors regarding the study, stressing the voluntary and non-obligatory nature of participation. All participants received guidance regarding the study and signed a document concerning the terms of free and informed consent. Women who declined to participate were not penalized either directly or indirectly.

### Sample and data collection

We conducted a cross-sectional quantitative study of primary and secondary data. The sample comprise women imprisoned in the seven medium- or maximum-security prisons in Mato Grosso do Sul, situated in the municipalities of Campo Grande, Corumbá, Jateí, Ponta Porã, Rio Brilhante, São Gabriel do Oeste, and TrêsLagoas. In addition, the medical records of participants were analyzed, excluding those from the municipality of Campo Grande, because we did not receive authorization to analyze those records.

Data collection was for the period from October 2015 and March 2016 when the female population was 1,001 in the seven prisons. Although the study was geared toward the total population, a sample size calculation was done for situations of collective refusal or obstruction by the institutions’ administrations. We used the web software Raosoft Sample Size to identify that 278 imprisoned women needed to be included to achieve a significance level of 95% and a margin of error of 5%. This sample was distributed among the prisons as follows: 103 in Campo Grande, 38 in Corumbá, 21 in Jateí, 41 in Ponta Porã, 26 in Rio Brilhante, 11 in São Gabriel do Oeste, and 31 in TrêsLagoas.

Convenience sampling was used, with imprisoned women invited to participate individually or collectively, according to the particularities of each location. When invited, participants received detailed information about the survey. For those who agreed to participate, a consent form was read and signed in duplicate; one copy was filed in the medical records or kept under the responsibility of a member of the health team, as recommended by the management of each establishment, and the other was filed by a researcher's guard.

The overall number of interviews exceeded the required sample size: 158 women were interviewed in Campo Grande; 89, in Corumbá; 40, in Jateí; 77, in Ponta Porã; 46, in Rio Brilhante; 24, in São Gabriel do Oeste; and 76, in TrêsLagoas. The entire prison population was invited, and there were 452 refusals, 30 non-authorizations, 7 exclusions because of cognitive difficulties, and 1 exclusion because of hospitalization. One participant was excluded because they had been included in the research in another prison. In total, 352 medical records were analyzed, excluding those in the municipality of Campo Grande, because that local administration denied access to the medical records.

The interview questionnaire was elaborated with semistructured questions that covered their sociodemographic profile (e.g.,nationality, state or country of residence, age, length of imprisonment, skin color, and educational level), their gynecological and obstetric profile, and their access to screening, information, and treatment for cervical cancer before and after being imprisoned. The form for obtaining information from the medical records was adapted from that used by Farias [19], which was designed to ascertain the imprisoned women’s access to control of cervical cancer, and included questions on tests, consultations, and treatment offered.

The information was recorded in forms elaborated in the Epi Info program, version 7.1.5, and was stored in Microsoft Office Excel 2013.

### Data analysis

Correlations were assessed between receiving the Pap test, the presence of cellular alterations, and if appropriate treatment was given. Appropriate treatment was considered to have been provided if the women were treated in accordance with the Brazilian Directives for Screening of Cervical Cancer (*Diretrizes Brasileiras para o Rastreamento do Câncer do Colo do Útero*) (BRASIL, 2011). A total of five possible responses were considered: 1) they had not undergone a Pap test in prison or outside prison; 2) they had undergone a Pap test, and if informed of the result, whether they received appropriate treatment; 3) they had undergone the Pap test and had been informed of the result, but did not receive treatment; 4) if they had undergone the Pap test and had not been informed of the result and had not received treatment; and 5) had undergone the Pap test and had not been informed of the result but had received treatment.

The data were analyzed using IBM SPSS, Version 23.0 (IBM Corp., Armonk, NY, USA) at a significance level of 5%. Associations were analyzed by the chi-squared test with the Bonferroni correction, as necessary. For the variables obtained in interviews, Pap test uptakes in the prisons were compared by age range, municipality, and length of imprisonment. For the variables obtained through analysis of medical records, the associations were analyzed between Pap tests in prisons in the municipality.

## Results

The sociodemographic characteristics of the imprisoned women in Mato Grosso do Sul are shown in Table 1. The analysis of race/skin color evidenced that being of mixed European/African descent was most frequent, with 324 (63.5%) women of mixed race. Most (n = 286; 56.1%) had studied for fewer than nine years.

**Table 1:** Sociodemographic characteristics of 510 incarcerated women in Mato Grosso do Sul between 2015 and 2016.

Variable	n (%)
**Municipality of the Prison Establishment**	
Campo Grande	158 (31.0)
Corumbá	89 (17.5)
TrêsLagoas	76 (14.9)
Ponta Porã	77 (15.1)
Rio Brilhante	46 (9.0)
Jateí	40 (7.8)
São Gabriel do Oeste	24 (4.7)
**Race**	
Mixed black/white	324 (63.5)
White	141 (27.6)
Black	34 (6.7)
Indigenous	8 (1.6)
Asian	1 (0.4)
Not stated	1 (0.2)
**Nationality**	
Brazilian	498 (97.6)
Others	12 (2.4)
**State or Country of Residence**	
Mato Grosso do Sul	354 (69.4)
Others	156 (30.7)
**Educational Level**	
None	2 (0.4)
Basic education–incomplete	286 (56.1)
Basic education–complete	46 (9.0)
Senior high school–incomplete	78 (15.3)
Senior high school–complete	74 (14.5)
Higher education–incomplete	14 (2.7)
Higher education–complete	10 (2.0)
**Age Range**	
18 to 24 years	158 (31.0)
25 to 34 years	207 (40.6)
35 to 44 years	98 (19.2)
45 to 54 years	30 (5.9)
55 to 64 years	14 (2.7)
65 years or older	3 (0.6)
**Length of imprisonment**	
0 to 1 month	44 (8.6)
2 to 12 months	251 (49.2)
13 to 24 months	107 (21.0)
25 to 36 months	64 (12.5)
37 months or older	44 (8.6)

Table 2 contains information about the interviewees’ consumption of tobacco and drugs: 264 (51.8%) were current smokers and 168 of these (63.6%) smoked between 10 and 20 cigarettes per day; and 283 (55.5%) engaged in illicit drugs use before imprisonment, with 226 (79.9%) doing so daily.

**Table 2:** Tobacco and drug use among 510 incarcerated women in Mato Grosso do Sul between 2015 and 2016.

Variable	n (%)
**Tobacco**	
Prior/past	121 (23.7)
Current	125 (24.5)
Prior/past	264 (51.8)
**Tobacco–daily frequency**(units of cigarettes)	
1 a 10	121 (45.8)
11 a 20	103 (39.0)
21 a 30	8 (3.0)
31 a 40	22 (8.3)
41 or more	10 (3.8)
**Drugs prior to imprisonment**	
Yes	283 (55.5)
No	227 (44.5)
**Drugs–daily frequency prior to imprisonment** (time per day)	
1 to 10	161 (56.9)
11 to 20	33 (11.7)
21 to 30	3 (1.1)
31 to 40	2 (0.7)
More than 40	2 (0.7)
Did not answer	82 (29.0)

Of the interviewees, 460 (90.2%) underwent menarche when aged between 10 and 15 years old, and 337 (66.1%) had their first sexual relations between the ages of 10 and 15 years old. Moreover, 266 (52.2%) had had between 1 and 3 pregnancies, 172 (33.7%) had had between 4 and 9 pregnancies, and 5 (1.0%) had had 10 or more pregnancies. It is important to note that not all women with a previous history of gestation, according to self-report, had term pregnancies, with several having spontaneous or induced abortions. In total, 298 (58.4%) had had between one and three births, and 339 (66.5%) denied a history of miscarriage or abortion, 166 (32.5%) reported having had 1 to 3 abortions, and 5 (1.0%) reported having had 4 to 9 abortions. The contraceptive pill was used by 339 women (66.5%), but that this was discontinued after being imprisoned (the reason for discontinuation was not included in the interview instrument). Still, 455 (89.2%) stated that they had used condoms in their sexual relations.

Table 3 shows that 292 women (57.3%) mentioned gynecological problems, of whom 135 (46.2%) mentioned infections as the most frequent problem. The interviews indicated that most women (n = 427; 83.7%) had had a Pap test before imprisonment, and that this was in the last 5 years for 304 (71.2%). Of the women who had received a Pap test, 298 (69.8%) stated that they had no cellular alterations at the time, while 70 (16.4%) reported alterations, of whom 42 (60.0%) mentioned infection as the most frequent alteration. Of those who reported alterations, 62 (88.6%) stated that they received treatment, and 56 (90.3%) received medication.

**Table 3:** Gynecological changes and PapTest uptake among 510 women before imprisonment (self-reported) in Mato Grosso do Sul between 2015 and 2016.

Variables	n (%)
**Gynecological Problem**	
Yes	292 (57.3)
No	210 (41.2)
Does not know	8 (1.6)
*Type of Problem	
Infections	135 (46.2)
Others	104 (35.6)
Alterations in the cervix	35 (12.0)
Sexually transmitted disease	14 (4.8)
Human papilloma virus	8 (2.7)
Cervical cancer	1 (0.3)
**Undertook Papanicolaou Test**	
Yes	427 (83.7)
No	83 (16.3)
**Collection Period**	
From 1999 to 2005	8 (1.9)
From 2006 to 2010	38 (8.9)
From 2011 to 2015	304 (71.2)
Does not remember	77 (18.0)
**Alteration**	
Yes	70 (16.4)
No	298 (69.8)
Does not know	55 (12.9)
Does not remember	4 (0.9)
*Type of Alteration	
Infections	42 (60.0)
Alterations in the cervix	12 (17.1)
Others	8 (11.4)
Inflammation	7 (10.0)
Human papilloma virus	2 (2.9)
**Undertook Treatment**	
Yes	62 (88.6)
No	7 (10.0)
Not answered	1 (1.4)
*Type of Treatment	
Medication	56 (90.3)
Biopsy	1 (1.6)
Cauterization	6 (9.7)
Others	1 (1.6)

*There could be one or more responses.

Among the gynecological problems mentioned by the interviewees, one woman (0.3%) mentioned having had cervical cancer and having undergone hysterectomy, radiotherapy, and brachytherapy to treat the disease. Among the 35 women (12.0%) who stated that they had had some type of cervical alterations, 1 woman (0.3%) mentioned having had a hysterectomy because of grade I cervical intraepithelial neoplasia (CIN I), which was confirmed in her medical records based on surgical biopsy with an International Classification of Diseases (ICD) code “C53.9–Malignant neoplasm of cervix uteri, unspecified”.

As shown in Table 4, half of the interviewed women (255) stated that they had been tested in prison, with 215 (84.3%) being tested in the current establishment. Of those who had been tested, 178 (69.8%) stated testing was done in 2015, and 134 (52.5%) reported that they did not know their result. Of those who had not had the Pap test in prison, 149 (58.4%) gave lack of opportunity as the reason.

**Table 4:** Uptake of the Papanicolaou Test in the current prison among 510 women in Mato Grosso do Sul between 2015 and 2016.

Variables	n (%)
**Undertook Papanicolaou Test**	
Yes	255 (50.0)
No	255 (50.0)
**Year of Collection**	
2015	178 (69.8)
2016	49 (19.2)
Other years	17 (6.7)
Does not remember	8 (3.1)
Did not answer	3 (1.2)
**Why the Woman Did Not Undertake the Test**	
Lack of opportunity	149 (58.4)
Chosen otto	36 (14.2)
Impossible to take a specimen	28 (11.0)
Does not know	16 (6.3)
**Place Where Test Undertaken**	
Prison unit	215 (84.3)
Others	40 (15.7)
**Abnormalities**	
Yes	20 (7.8)
No	101 (39.7)
Does not know	134 (52.6)
**Information on the Abnormality**	
Yes	19 (95.0)
Does not know	1(5.0)
**Undertook Treatment**	
Yes	90.0 (18)
No	5.0 (1)
Does not know	5.0 (1)
**Type of Treatment**	
Medication	16 (88.9)
Cauterization	1 (5.6)
Did not answer	1 (5.6)
**Place of Treatment**	
Prison unit	94.44 (17)
Health unit	5.56 (1)
**Treatment Situation**	
Treatment completed	12 (66.7)
Treatment not completed	4 (22.2)
Treatment on going	1 (5.6)
Does not know how to answer	1 (5.6)
**Reason for Treatment Not Completed**	
Did not repeat the test	2 (50.0)
By choice	1 (25.0)
Lack of medication prescribed	1 (25.0)

A total of 20 participants (7.8%) who had a Pap test said that their result had been abnormal. Among these, 19 stated that they received information regarding the type of abnormality, 18 received treatment, and 17 were treated in the prison (medications and vaginal cremes predominated). Of the women who received treatment, 12 (66.7%) stated that they had completed the treatment and 4 (22.2%) that they had not completed the treatment. Two of those who had not finished treatment had not done so because the test was not repeated, one had not been prescribed medication was, and one chose to stop treatment.

Table 5 shows evidence of an association between the Pap test and age group (p<0.01), with the greatest test uptake among women aged 35–64 years old, and less uptake in the groups aged 18–24 years and 65 years and older. Further differences were observed in Pap test uptake between prisons indifferent municipalities (p<0.01), and there was greater uptake among women imprisoned for 13 months or more when compared with those imprisoned for fewer than 12 months.

**Table 5:** Age range, municipality, length of imprisonment, and Papanicolaou Test uptake in prison among 510 women in Mato Grosso do Sul between 2015 and 2016.

Variable	Papanicolaou Test in the prison establishment		P value
	Yes	No	
**Age Range** (years old)			
18 to 24	55 (34.8)	103 (65.2)	<0.01
25 to 34	113 (54.6)	94 (45.4)	
35 to 44	56 (57.1)	42 (42.9)	
45 to 54	21 (70.0)	9 (30.0)	
55 to 64	10 (71.4)	4 (28.6)	
65 years old or over	0 (0.0)	3 (100.0)	
**Municipality**			
Rio Brilhante	42 (91.3)	4 (8.7)	<0.01
São Gabriel do Oeste	20 (83.3)	4 (16.7)	
Jateí	24 (60.0)	16 (40.0)	
Campo Grande	81 (51.3)	77 (48.7)	
TrêsLagoas	38 (50.0)	38 (50.0)	
Corumbá	32 (36.0)	57 (64.0)	
Ponta Porã	18 (23.4)	59 (76.6)	
**Length of imprisonment** (month)			
0 to 1	6 (13.6)	38 (86.0)	<0.01
2 to 12	88 (35.1)	163 (64.9)	
13 to 24	78 (72.9)	29 (27.1)	
25 to 36	46 (71.9)	18 (28.1)	
37 months or over	37 (84.1)	7 (15.9)	

The results are presented in relative frequency (absolute frequency). P value in the chi-squared test. (chi-squared test, p<0.05, with the Bonferroni correction).

Associations were not found between abnormalities in the Pap test in prison and smoking (p = 0.261), use of the contraceptive pill (p = 0.733), coitarche (p = 0.738), pregnancy (p = 0.575), and parity(p = 0.859).

Of the 352 medical records analyzed, 211 (59.9%) did not contain information regarding the Pap test or treatment. Another 129 (36.6%) contained a record of the undertaking of the test, and 102 (79.1%) of these were done in 2015. In 12 medical records (3.4%) there was a record of not undertaking tests for other reasons.

Of the 129 medical records that contained information about collecting the Pap test specimen, 110 (85.3%) had a record of the results, with 104 (94.5%) negative results for neoplasm and 3 (5.5%) showing some type of cellular alteration. Among the results with alterations, 3 (50.0%) had "atypical squamous cells of undetermined significance (ASC-US)"; 2 (33.3%) had "low-grade squamous intraepithelial lesions (LSIL)"; and 1 (16.7%) had "atypical glandular cells of undetermined significance, cannot exclude high-grade intraepithelial lesion (AGC)."These records were from the in Ponta Porã (n = 2; 33.3%), Rio Brilhante (n = 2; 33.3%), Jateí (n = 1; 16.1%) and São Gabriel do Oeste(n = 1; 16.1%). One woman with a result of ASC-US received medical advice and repetition of the test, a woman with LSIL was vaccinated against HPV, another had no recorded treatment, and although another woman with AGC was referred to secondary care for biopsy, colposcopy, and conization, there was no record of undertaking these interventions. There was an association between uptake of the Pap test in prison establishments in different municipalities according to the medical records (p<0.01).

## Discussion

We studied Pap test uptake for the screening and early detection of cervical cancer among imprisoned women in Mato Grosso do Sul. Overall, the results showed that screening was provided in a non-systematic way, with significant differences in practice between prisons (p <0.001). Although the interviewees often reported having had a test, few of the analyzed records contained information about screening, making it difficult to consolidate the regular and continuous health care for this population.

For the year 2012, there were 528,000 new cases of cervical cancer and 266,000 deaths worldwide. For each ten deaths from the disease, approximately nine occurred in developing countries, reflecting failures in screening and early detection that result in late diagnosis and reduced survival [20–21]. It should be noted that women who are in prison have fewer opportunities for cervical cancer screening; this is important because it is not only the most common cancer in this population, but because it is also associated with high rates of abnormalities in this population [3].

In this study the women had become sexually active before the age of 15 years, most had used tobacco, and most had used illicit drugs, indicating high rates of major risk factors for developing cervical cancer.

Although there were no significant associations between educational level and Pap test uptake before or after imprisonment, it was striking that most women had not completed basic school education. This is important because low educational level is a social risk factor for cervical cancer, in that it intensifies the individual and collective vulnerabilities to which these women are exposed [15]. Educational level is directly related to the level of health education, as poorly educated individuals have difficulty understanding information and advice, whether in written or verbal form. In turn, this influences their behavior in relation to health and to the prevention of diseases and health problems. Studies indicate that women with higher levels of education are more likely to have Pap tests [22].

The prevalence of tobacco use was high in this study, with rates exceeding 75.0%. Tobacco use is known to be more frequent in prison populations in comparison with the general population, with rates of 50.0%–83.0% being common [23]. However, the number of women who mentioned tobacco use (51.8%) is comparable to that in other studies. For example, a study in Brazil indicated that 67.1% of women prisoners were smokers [15], compared with other studies showing rates of 58.2% in Spain and 46.7% in the United States [23,24]. This is concerning given tobacco’s known carcinogenic potential. Studies have shown that some substances found in cigarettes, including their metabolites, are present in the cervical mucus of women who smoke; it is posited that this may cause a reduction in the quantity of Langerhans cells, which help defend the epithelial tissue from damage, in these women’s cervixes. Tobacco also seems to influence the natural history of infection by HPV, which is the main risk factor for the disease [15,24,25].

The predominance of sexual activity starting between the ages of 10 and 15 years (66.1%) reflects another important risk factor for developing the disease. This is a well-established risk caused by the potential for harm to the as-yet immature cervix, which can be left more susceptible to sexually transmitted infections, and thereby, to infection by HPV [15–26–27–28]. Early initiation of sexual activity is particularly associated with an increase in the risk of infection by genotype 16 of the virus [27].

Few of the imprisoned women (13.7%) reported never having been pregnant, and most (86.9%) reported having had one or more pregnancy. This is similar to the result of another study by Nicolau *et al*. (2015) in which 80.6% of imprisoned women in Brazil had had one or more pregnancy. In developing countries, there is an association between pregnancy and cervical cancer [29]. It is suggested that there is a relationship between the levels of estrogen and progesterone during the gestational period, and that this modifies the cervical squamo columnar junction, causing the transformation zone to remain in the ectocervix for some time, favoring exposure to HPV and contributing to the persistence of infection and to the progression of associated lesions [30]. Another important factor is the suppression of the immune system that occurs during pregnancy, which may contribute to HPV replication [29].

In one study of pregnant women in Brazil who were not imprisoned, the prevalence of HPV was 25.3%. By contrast, different results were found in another Brazilian study, albeit in a smaller sample, which did not find an association between pregnancy, HPV infection, and cervical lesions. In Europe, among women who were not in prison, associations have been evidenced between the number of births and the risk for developing cervical intraepithelial neoplasia grade 3 and carcinoma in situ, without there being a direct relationship with invasive cervical cancer [29,30,31].

Regarding oral contraceptive use, a significant proportion of the sample (66.5%) mentioned use. This is consistent with the finding by Anjos *et al*. (2013), who reported a rate of 70.5% in imprisoned women. Some participants reported during interview that they had used the method before imprisonment, but that this had stopped since imprisonment. The association between oral contraceptive use and the risk of developing cervical cancer and its precursor lesions may increase with the length of use, but may also reduce if their use is interrupted [30]. As a result, the significant percentage of imprisoned women who had previously used or were currently using oral contraceptives constitutes relevant information that needs to be considered when assessing any potential reduction in the risk of developing cervical cancer.

In this study, the proportion of women with cervical cancer was similar to that reported in other studies. In Brazil, it was estimated that approximately 16,340 new cases occurred in 2016, with a risk estimate of 15.85 cases per 100,000 women; however, no information is available for the prison population [11]. In the United States, approximately 12,000 new cases of cervical cancer are diagnosed each year, and it has been well-documented that the prison population is at greater risk of certain chronic health conditions, including cervical cancer. Indeed, in women aged 40 years old or over, the risk is four to five times greater in the prison population compared with the free population [32–33].

Abnormalities in the Pap test were mentioned by a few interviewees, with infection being most common. This conflicts with the findings in a report by Binswanger *et al*. (2011) [34], in which there was a high prevalence of abnormal Pap test results. In the analysis of medical records in our study, a few women (5.5%) had records of cellular changes from the Pap test, which again conflicts with other studies that have revealed higher proportions (e.g., 10.6%) among all tests and rates that are 6 to 11 times greater in imprisoned women than in non-imprisoned women [33,35]. However, this may reflect poor documentation in the clinical records in this study.

We note that ignorance of test results among women and a lack of detail in the medical records are issues that require urgent attention. When tests are done, we found that not only are women not told of the results but also that the findings are frequently left unrecorded. This indicates a need to train professionals who provide care in prisons. Of equal importance, we found that women may receive appropriate treatment, but that there was often failure to inform them about the results. This may indicate a lack of educational activities and information being provided about health and well-being. Such activities could prepare women for return to the community, which is important given the short periods for which most are imprisoned.

Significant associations were found between Pap test uptake in prison and age, with peak uptake in those older than 25 years. This broadly indicates compliance with the target population stipulated by the Ministry of Health in Brazil [12]. Pap test uptake was lower in women aged younger than 25 years, indicating less attention to recommendations regarding this population [36]. However, according to Sasiene *et al*. (2009), screening women aged from 20 to 24 years old does not significantly affect the incidence of cervical cancer developing before the age of 30 years [37].

Despite the presence of national directives for cervical cancer screening since 2011 [12], half of the women interviewed mentioned not having had a Pap test in prison. Statistical analysis indicated that there was an association between length of imprisonment and Pap test uptake, with test uptake being more likely among women imprisoned for longer periods. This indicates a possible failure to maintain a standard of care for cervical screening within the prisons studied. Studies indicate that women prisoners should have the same access to health services as women in the general population [38]. The most recent declaration by the World Health Organization in Europe indicates the need for gynecological care at regular intervals, with a guarantee of diagnosis and treatment for complications [39]. The Pap test remains an excellent test for screening cervical cancer because of its low cost, ease of application, and high sensitivity; consequently it is included in international recommendations for the health of female populations [14]. Nevertheless, there is a need to establish regulated international minimum standards for use in the female prison population [38]. This includes the need to hold and maintain individual medical records.

Two positive findings of this study were the large number of interviewed women (83.7%) who had had a Pap test while not in prison, and the fact that88.6% of those who had abnormal results received treatment. These results reflect adherence with the guidelines for cervical screening in the female population. It was also observed that many women in prison received treatment based on their smear results when the treatment was medication-based. However, the one woman who required treatment that was more complex had no record of her outcome, suggesting the need to transfer healthcare record keeping to the health system.

This study has some limitations. Women were interviewed and we did not have access to all medical records, which is a major problem because it limits our understanding of the continuity of care and interventions in the healthcare network in primary and specialized care. Also, the retrospective record review, the lack of records, and the presence of incomplete records make it difficult to draw conclusions for the evaluation and proposal of public policies.

## Conclusions

The study indicated that Pap tests were not done in a regular or systematic manner. Similarly, medical records were present for most women, but they typically contained incomplete information, and they were not systematically maintained when women received assistance from the prison or from other healthcare establishments. The lack of adequate records is worrying when seeking to formulate and evaluate health policies in this population, because such records are the primary source of information used for decision making. Medical records could enable continuous care by providing care networks that are more efficient. Research is needed to investigate the reasons for the absence of records.

## Supporting information

S1 File Interview questionnaire. (PDF)

S2 File Medical records formulary. (PDF)

S3 File Database interview questionnaire. (XLSX)

S4 File Database medical records. (XLSX)
