# Supplementary material for: Screening for cervical cancer in imprisoned women in Brazil
Source: PLoS One. 2017 Dec 18;12(12):e0187873. doi: 10.1371/journal.pone.0187873 (PMC5734681; doi:10.1371/journal.pone.0187873)
Supplement: S1 File — (PDF) [file pone.0187873.s001.pdf]

INSTRUMENT FOR DATA COLLECTION OF THE ENCARCERATED'S WOMEN

County:
Date:
Prison unit:
Women's order number:

I: Characterization of the internal:

01 - Age ______ years old.

02 - Race / color:
a. ( ) White
b. ( ) Brown
c. ( ) Black
d. ( ) Yellow
e. ( ) Indigenous

03 - Nationality: ______________________

04 - Municipality of residence before prison: _________________________

05 - Time in confinement (years, months): ________________

06 - Marital Status:
a. ( ) Married
b. ( ) Decided or judicially separated
c. ( ) Divorced
d. ( ) Widow
e. ( ) Single
f. ( ) Stable union

07 - Degree of education:
a. ( ) Uneducated
b. ( ) Incomplete elementary school
c. ( ) Complete primary education
d. ( ) Incomplete secondary school
e. ( ) Complete high school
f. ( ) Incomplete higher
g. ( ) Graduated

08 - Previous occupation: _________________________

09 - What is the income of your entire family (add up all the gross wages, without deductions from the people of your working family group, including your income _________________ (in reais).

10 - How many people depend on this income (only those who live in the house, including you)? __________________.

11–Do you visit your family / friends?

a. (   ) Yes
b. (   ) No
If so, how often? ________________________

12–Do you receive intimate visits?
a. (   ) Yes
b. (   ) No
If so, how often? ________________________

II–Personal history:

13–Smoke:
a. (   ) Make use
b. (   ) Already make use
c. (   ) Never make use
If so, how many cigarettes per day? _______________

14–Have you ever used any illicit drugs?
a. (   ) Yes
b. (   ) No
If yes, how often in the year, month or week (1, 2, 3, 4, 5,…, how many times)?______.

III - Gynecological and obstetric history:

15 - Menarche (in years): ______.

16 - Coitarca (in years): ______.

17 - Gestation _______ For_______ Abortion________

18 - Contraceptive pill:
a. (   ) Make use
b. (   ) Already make use
c. (   ) Never make use

19–Condom in sexual relations:
a. (   ) Make use
b. (   ) Already make use
c. (   ) Never make use

20–Have you ever had any gynecological problems?
a. (   ) Yes
b. (   ) No
c. (   ) She doesn’t know
If yes, what was the problem?____.

21 - Have you heard about cervical cancer screening?
a. (   ) Yes
b. (   ) No

22–Do you know the importance of performing cervical cancer screening?
a. ( ) Yes
b. ( ) No

23–Before you go to prison, have you ever had a cervical cancer screening?
a. ( ) Yes
b. ( ) No
c. ( ) Cannot remember
If yes, when (year) ___________

24–Has there been any change in the test result?
a. ( ) Yes
b. ( ) No
c. ( ) She doesn’t know
d. ( ) Cannot remember
If yes, what type of alteration?______________________________

25–If the answer to the previous question is yes, performed treatment?
a. ( ) Yes
b. ( ) No
c. ( ) She doensn’t know
d. ( ) Cannot remember
If yes, Which treatment?_____________________________

IV: Characterization of health care related to the Control of Cervical Cancer:

26–When you entered this Prison unit, were you asked if you had already taken the cervical cancer screening test?
a. ( ) Yes
b. ( ) No
c. ( ) Cannot remember

27–Have you performed the collection of the preventive examination after arrival at the prison unit?
a. ( ) Yes
b. ( ) No
c. ( ) Cannot remember
If yes, When (year) _____________

28–If the answer to the previous question is no, why didn’t performed preventive examination?
____
____
____

29 - Was the collection of the preventive examination performed after arrival at the prison unit, where the examination was carried out?
a. ( ) Prison unit
b. ( ) Health unit

c. ( ) Others: ___________________

30–Has there been any change in the test result?
a. ( ) Yes
b. ( ) No
c. ( ) Cannot answer

31–If yes, did you receive information about the change identified in the preventive examination?
a. ( ) Yes
b. ( ) No
c. ( ) Cannot answer

32–Has any treatment been performed because the alteration presented in the preventive exam?
a. ( ) Yes
b. ( ) No
c. ( ) Cannot answer

If yes, what treatment (periodicity of consultations and type of treatment received)?
____
____
____
____

33–If any treatment was performed, where was it performed?
a. ( ) Prison unit
b. ( ) Health unit
c. ( ) Center of Specialties
d. ( ) Others. Which are:____

34–What is the treatment situation?
a. ( ) It was finalized
b. ( ) Not finalized
c. ( ) It is treating
d. ( ) Cannot answer

35–If the answer to the previous question is interrupted treatment, can you tell why the treatment was interrupted?
____
____
____
____
