# Supplementary material for: Screening for cervical cancer in imprisoned women in Brazil
Source: PLoS One. 2017 Dec 18;12(12):e0187873. doi: 10.1371/journal.pone.0187873 (PMC5734681; doi:10.1371/journal.pone.0187873)
Supplement: S2 File — (PDF) [file pone.0187873.s002.pdf]

INSTRUMENT FOR DATA COLLECTION OF THE ENCARCERATED'S WOMEN MEDICAL RECORDS

County:
Date:
Prison unit:
Women's order number:

<table>
<tr><td colspan="3">I: Characterization of the internal</td></tr>
<tr><td colspan="3">01- Age ______ years old.</td></tr>
<tr><td colspan="3">II: Characterization of health care related to the Control of Cervical Cancer</td></tr>
<tr><td colspan="3">02–The oncotic colpocitology examination was collected after the arrival of the woman in the prison unit:
a. (   ) Yes
b. (   ) No
c. (   ) No record information
If yes, when (year)</td></tr>
<tr><td colspan="3">03–Place where the collection was made after the arrival of the woman in the Prison unit:
a. (   ) Prison unit
b. (   ) No record information
c. (   ) Others:</td></tr>
<tr><td colspan="3">04–Record of the results of the oncotic colpocytology examination:
a. (   ) Yes
b. (   ) No
c. (   ) No record information</td></tr>
<tr><td colspan="3">05–Result of oncotic colpocytology examination - Within the limits of normality in the material examined:
a. (   ) Yes
b. (   ) No
c. (   ) No record information</td></tr>
<tr><td colspan="3">06 - According to the alteration presented in the result of the oncotic colpocytology examination, which was performed:</td></tr>
<tr><td>1</td><td>Atypical squamous cells of undetermined significance, possibly non-neoplastic (ACU-US)</td><td>a. (   ) Repeats the cytopathological examination in 06 months.
b. (   ) Repeats the cytopathological examination in 12 months.
c. (   ) Forwards to other levels of health care.
d. (   ) No record information
e. (   ) Others:________________________</td></tr>
<tr><td>2</td><td>Células escamosas atípicas de significado indeterminado, quando não se pode excluir lesão intraepitelial de alto grau (ASC-H)</td><td>a. (   ) Repeats the cytopathological examination in 06 months.
b. (   ) Repeats the cytopathological examination in 12 months.
c. (   ) Forwards to other levels of health care.
d. (   ) No record information
e. (   ) Others:_______________</td></tr>
<tr><td>3</td><td>Atypical glandular cells of undetermined significance, possibly non-neoplastic or</td><td>a. (   ) Repeats the cytopathological examination in 06 months.
b. (   ) Repeats the cytopathological examination</td></tr>
</table>

	when high-grade intraepitheliallesion cannot be excluded (AGS).	in 12 months.<br>c. ( ) Forwards to other levels of health care.<br>d. ( ) No record information<br>e. ( ) Others:________________________
4	Atypical cells of indefinite origin	a. ( ) Repeats the cytopathological examination in 06 months.<br>b. ( ) Repeats the cytopathological examination in 12 months.<br>c. ( ) Forwards to other levels of health care.<br>d. ( ) No record information<br>e. ( ) Others:________________________
5	Low-gradesquamous intraepithelial lesion (LSIL)	a. ( ) Repeats the cytopathological examination in 06 months.<br>b. ( ) Repeats the cytopathological examination in 12 months.<br>c. ( ) Forwards to other levels of health care.<br>d. ( ) No record information<br>e. ( ) Others:________________________
6	High-grade intraepithelial lesion (HSIL)	a. ( ) Repeats the cytopathological examination in 06 months.<br>b. ( ) Repeats the cytopathological examination in 12 months.<br>c. ( ) Forwards to other levels of health care.<br>d. ( ) No record information<br>e. ( ) Others:________________________
7	High-grade intraepithelial lesion not being able to exclude microinvasion or invasive squamous cell carcinoma or clinical suspicion of invasion.	a. ( ) Repeats the cytopathological examination in 06 months.<br>b. ( ) Repeats the cytopathological examination in 12 months.<br>c. ( ) Forwards to other levels of health care.<br>d. ( ) No record information<br>e. ( ) Others:________________________
8	Adenocarcinoma in situ (AIS) and invasive	a. ( ) Repeats the cytopathological examination in 06 months.<br>b. ( ) Repeats the cytopathological examination in 12 months.<br>c. ( ) Forwards to other levels of health care.<br>d. ( ) No record information<br>e. ( ) Others:________________________

07–Treatment for the woman who presented the result of the altered colpocytological examination:
a. ( ) Yes
b. ( ) No
c. ( ) No record information

08–There was a referral from the woman:
a. ( ) Yes
b. ( ) No
c. ( ) No record information

09–If the woman was referred to what level of attention:
a. ( ) Primary care level

b. ( ) Secondary care level<br>c. ( ) Tertiary care level
10–If the woman was referred with a result of the altered colpocytological examination to another health care service, there is a record of whether the team received information about the behaviors adopted for the treatment of women:<br>a. ( ) Yes<br>b. ( ) No<br>c. ( ) No record information
11–If the answer to the previous question is yes, what were the types of conduct adopted:<br>a. ( ) Colposcopy<br>b. ( ) Biopsy<br>c. ( ) Conization<br>d. ( ) Exercise of Transformation Zone (EZT)<br>e. ( ) Hysterectomy<br>f. ( ) Forwarding to the tertiary unit<br>g. ( ) Others
12 - Treatment:<br>a. ( ) It was finalized<br>b. ( ) Not finalized<br>c. ( ) It is treating<br>d. ( ) No record information
